# Neonatal Severe Hyperparathyroidism Causing Life-Threatening Hypercalcemia Treated With Medical and Surgical Management

**DOI:** 10.1210/jcemcr/luae133

**Published:** 2024-08-09

**Authors:** Kerri Rosettenstein, Andrew Parasyn, Kristen Neville, Shihab Hameed

**Affiliations:** Department of Paediatric Endocrinology and Diabetes, Sydney Children's Hospital Randwick, Sydney, 2031, Australia; School of Paediatrics and Child Health, University of New South Wales, Sydney, 2031, Australia; Department of Paediatric Endocrinology and Diabetes, Royal North Shore Hospital, Sydney, 2065, Australia; Department of Paediatric Endocrinology and Diabetes, Sydney Children's Hospital Randwick, Sydney, 2031, Australia; Department of Surgical Oncology, Prince of Wales Hospital, Sydney, 2031, Australia; Department of Paediatric Endocrinology and Diabetes, Sydney Children's Hospital Randwick, Sydney, 2031, Australia; School of Paediatrics and Child Health, University of New South Wales, Sydney, 2031, Australia; Department of Paediatric Endocrinology and Diabetes, Sydney Children's Hospital Randwick, Sydney, 2031, Australia; School of Paediatrics and Child Health, University of New South Wales, Sydney, 2031, Australia; Department of Paediatric Endocrinology and Diabetes, Royal North Shore Hospital, Sydney, 2065, Australia; Northern Clinical School, University of Sydney, Sydney, 2065, Australia

**Keywords:** neonatal, severe, hyperparathyroidism, hypercalcemia, parathyroidectomy

## Abstract

A 3-day-old male presented to a peripheral remote hospital in New South Wales, Australia, with tachypnea. He was found to have hypercalcemia, with ionized calcium >2.5 mmol/L (>10 mg/dL) (0.97-1.5 mmol/L or 1.14-1.3 mg/dL) and serum calcium of 3.85 mmol/L (15.43 mg/dL) (2.2-2.8 mmol/L or 8.5-10.5 mg/dL). Peak serum calcium was 5.4 mmol/L (21.64 mg/dL). He was transferred to a tertiary pediatric intensive care unit. Medical management (including hyperhydration, diuretics, corticosteroids, bisphosphonates, cinacalcet, and calcitonin) failed to maintain normocalcemia; therefore, total parathyroidectomy was performed on day 16 of life. Hungry bones syndrome developed postoperatively, requiring high doses of calcium, calcitriol, and phosphate supplementation. Genetic testing identified compound heterozygosity for 2 likely pathogenic variants in the calcium-sensing receptor gene. He is now 3 years old and is growing and developing without any concerns. This case highlights the importance of aggressive initial management in addressing severe hypercalcemia through perioperative management principles as well as the prolonged nature of hungry bones syndrome.

## Introduction

Neonatal severe hyperparathyroidism (NSHPT) is a rare, life-threatening disorder, with only approximately 100 known reported cases (1). Newborns present in the first few days of life and are found to have severe hypercalcemia, hyperparathyroidism, osteopenia, and hypocalciuria. Clinically, babies can present with a variety of signs including respiratory distress, hypotonia, bony fractures or deformities, intestinal dysmotility, and failure to thrive. Most cases of NSHPT are associated with variants in the calcium-sensing receptor (CaSR), which is the predominant sensor of extracellular ionized calcium. Its role is to regulate PTH release from the parathyroid glands and calcium reabsorption from the renal tubules (2). Failure of the CaSR to recognize elevated serum calcium leads to unopposed PTH activity, causing skeletal demineralization, decreased renal calcium excretion, and severe hypercalcemia, which can be fatal (3).

Treatment of NSHPT involves IV hyperhydration, diuretics, and bisphosphonate therapy. More recently, cinacalcet (a calcimimetic) has been used to increase the responsiveness of the abnormal CaSR (2, 3). Subtotal/total parathyroidectomy can be offered.

## Case Presentation

A 3-day-old male presented to a rural hospital in New South Wales, Australia, with tachypnea and hypoxia (oxygen saturation, 91% in room air). He was the fourth child, born in good condition, to nonconsanguineous parents at full term, birth weight 3880 g (70th percentile). There was no family history of hypercalcemia, bone disease, or renal stones. He had been breastfed from birth.

## Diagnostic Assessment

**Figure 1.**
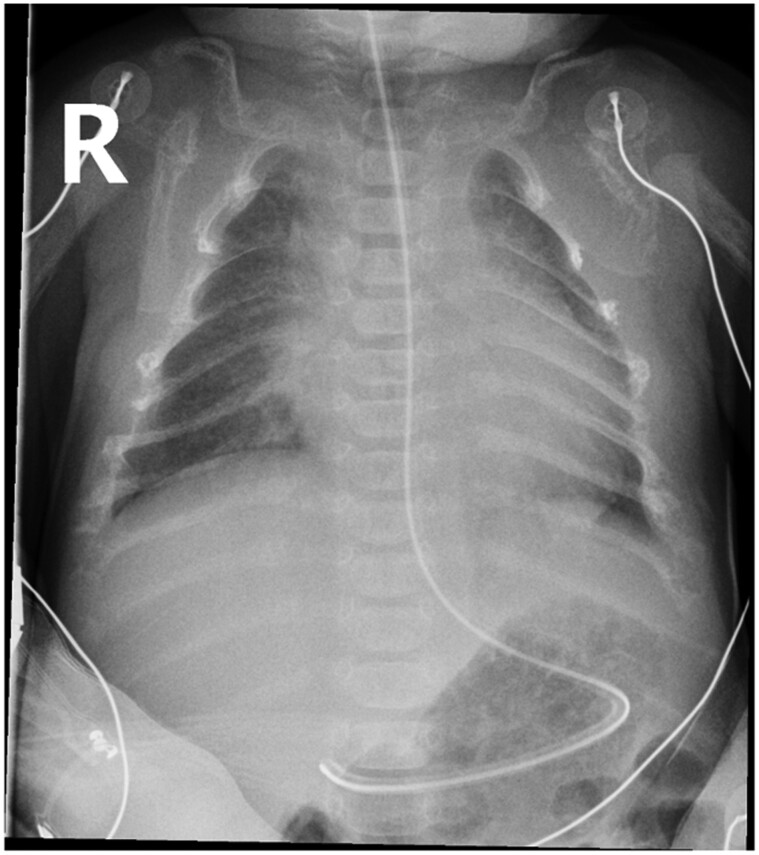
Chest x-ray at presentation showing diffuse osteopenia and coarsened trabeculae throughout the thoracic cage.

A chest x-ray showed marked osteopenia and coarsened trabeculae throughout the thoracic cage (Fig. 1). Venous blood gas showed an ionized calcium of >2.5 mmol/L (>10 mg/dL) (1.1-1.35 mmol/L or 4.5-5.3 mg/dL). Formal serum calcium was markedly elevated; 3.85 mmol/L (15.43 mg/dL) (2.2-2.8 mmol/L or 8.5-10.5 mg/dL), phosphate was reduced; 0.7 mmol/L (2.17 mg/dL) (1.45-2.5 mmol/L or 4.49-7.74 mg/dL), and PTH level was inappropriately elevated; 128 pmol/L (1707 pg/mL) (1.6-6.9 pmol/L or 7-59 pg/mL).

**Figure 2.**
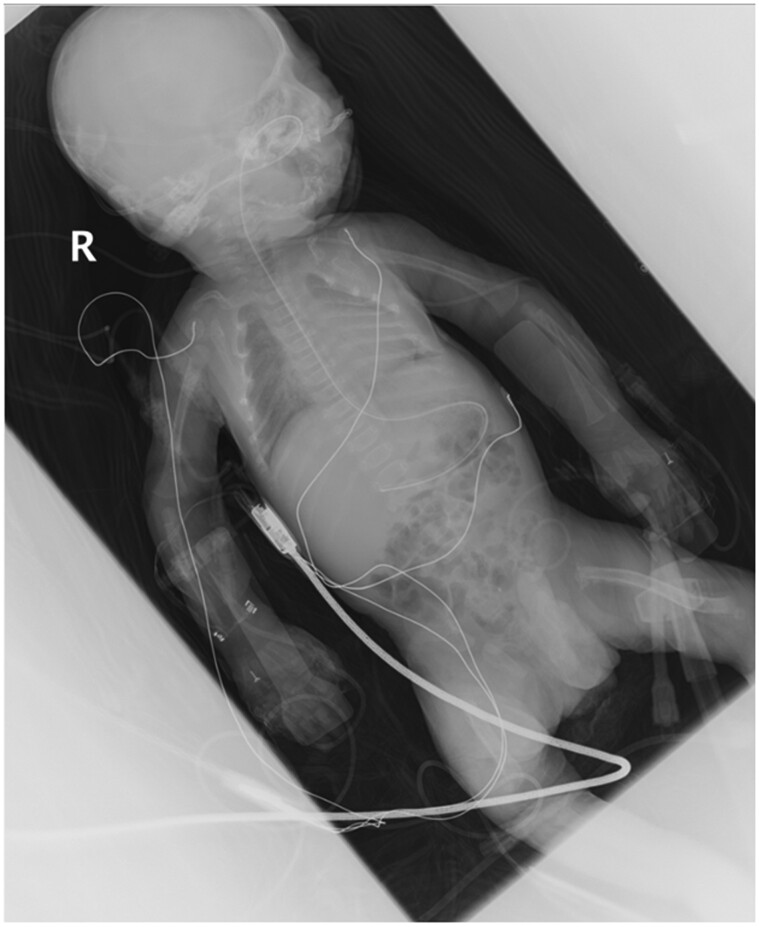
Babygram showing long bone bowing, most prominent in the femurs and probable rib fractures at 6th, 7th, 8^th^, and 9th ribs on the right, and 3rd, 4th, 7^th^, and 8th ribs on the left.

Following urgent transfer to our tertiary hospital intensive care unit (570 km [358 miles] away), a babygram showed long bone bowing (Fig. 2) and probable rib fractures. Antenatal ultrasound had similarly revealed a bowed left femur. Electrocardiogram was normal.

The blood and urine tests of both parents were consistent with a diagnosis of familial hypocalciuric hypercalcemia (Table 1), which was likely to represent heterozygosity for a CaSR abnormality.

**Table 1.** Blood and urine results for child's parents as well as the child

	Serum calcium	Serum creatinine	Urine calcium	Urine creatinine	PTH	Urine calcium creatinine ratio	Fractional excretion of calcium
Father	2.61 mmol/L (10.5 mg/dL)	124 µmol/L (1.4 mg/dL)	4.0 mmol/L (16 mg/dL)	19.0 mmol/L (214 mg/dL)	4.2 pmol/L (39.6 pg/mL)	0.2	0.01%
Mother	2.41 mmol/L (9.7 mg/dL)	69 µmol/L (0.8 mg/dL)	1.2 mmol/L (4.8 mg/dL)	13.7 mmol/L (155 mg/dL)	7.2 pmol/L (67.9 pg/mL)	0.1	0.0025%
Child	2.16 mmol/L (8.7 mg/dL)	28 µmol/L (0.3 mg/dL)	<0.2 mmol/L (<0.8 mg/dL)	0.6 mmol/L (7 mg/dL)	0.7 pmol/L (6.6 pg/mL)	<0.3	N/A

Normal range for serum calcium is 2.1-2.6 mmol/L (8.5-10.5 mg/dL). Normal range for serum creatinine differs according to sex and age—for adult males 60–110 μmol/L (0.7–1.2 mg/dL), adult females 45–90 μmol/L (0.5–0.9 mg/dL) and infant 17–50 μmol/L (0.2–0.6 mg/dL). Normal range for PTH is 1.6-6.9 pmol/L (7-59 pg/mL). Normal urine calcium to creatinine ratio is <0.8. Normal fractional excretion of calcium is >0.01%. Father's results indicate an inappropriately normal PTH for a slightly raised calcium and a low fractional excretion of calcium. Mother's results indicate an inappropriately high PTH for a normal calcium and a low fractional excretion of calcium. Both parent's results are consistent with a diagnosis of familial hypocalciuric hypercalcemia (FHH). Child's blood and urine were collected 20 days after parathyroidectomy. Fractional excretion of calcium is calculated as calcium mmol/L (urine)×creatinine µmol/L (serum)/calcium mmol/L (serum)×creatinine µmol/L (urine).

NSHPT was assumed to be the cause of his presentation based on the severity of hypercalcemia and parent biochemistry. This was confirmed 7 days after presentation by Trio Whole Genome Sequencing, showing compound heterozygosity for 2 likely pathogenic variants in the *CASR* gene (using American College of Medical Genetics and Genomics classification) on chromosome 3 (c. 190A > G; p.(Asn64Asp) and c. 101T > C; p.(Leu34Pro)).

Renal ultrasound showed no evidence of nephrocalcinosis. Ultrasound of the neck revealed 2 small soft tissue nodules at the inferior poles of the thyroid gland, consistent with parathyroid glands (left side, 5.1 × 3.1 × 3.3 mm; right side, 4.6 × 3.1 × 3.2 mm) and a normally appearing thyroid. Sestamibi scan did not identify the parathyroid or thyroid glands. Four-dimensional computed tomography scan confirmed rib fractures but did not identify a parathyroid gland or soft tissue abnormality.

## Treatment

**Figure 3.**
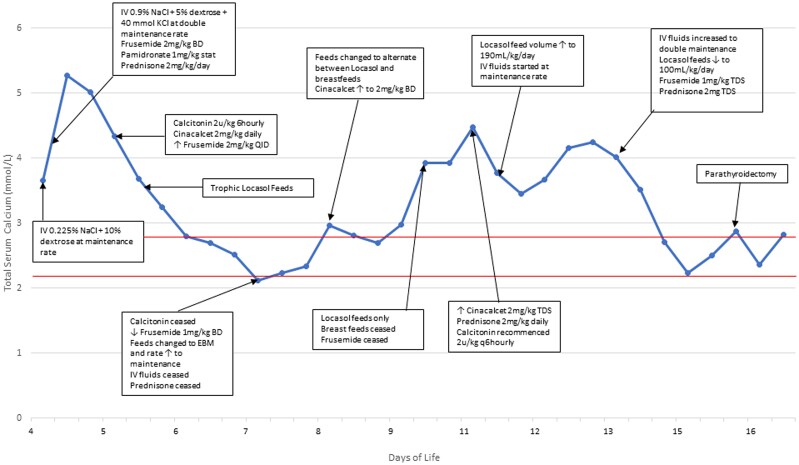
Impact of medical interventions on serum calcium levels from presentation on day 4 of life, to parathyroidectomy on day 16 of life. Normal serum calcium ranges from 2.2–2.8 mmol/L (indicated by the horizontal red lines).

On initial presentation, hypoxia was treated with 250 mL low-flow nasal prong oxygen. Initial fluid management was with IV 0.225% sodium chloride and 10% glucose at maintenance rate (16 mL/hour) but changed after hypercalcemia was identified to 0.9% sodium chloride with 5% glucose and 40 mmol potassium chloride at double maintenance for hyperhydration. Following pediatric endocrine consultation, a variety of measures were introduced to lower the serum calcium (Table 2 and Fig. 3). Frusemide was commenced at 2 mg/kg twice daily. A single dose of pamidronate 1 mg/kg (diluted as 10 mg in 100 mL of 0.9% sodium chloride and infused over 4 hours) was given within 6 hours of presentation. Prednisone 2 mg/kg/day was commenced. Despite these interventions, the serum calcium rose during transfer to a maximum of 5.4 mmol/L (21.64 mg/dL) (2.2-2.8 mmol/L or 8.82-11.22 mg/dL).

**Table 2.** Mechanism of action of medications used to treat hypercalcemia

Medical management	Mechanism of action
IV hyperhydration	Increases renal calcium excretion. Use of a higher saline concentration in IV fluids is thought to be beneficial as sodium assists in the cotransport of calcium excretion.
Frusemide	Facilitates diuresis and additional urine calcium excretion.
Calcitonin	Decreases bone mineral resorption by inhibiting osteoclast activity.
Prednisone	Decreases intestinal absorption of calcium, thereby increasing renal excretion.
Pamidronate	A bisphosphonate, which binds to the surface of bone and inhibits calcium release by interfering with osteoclast-mediated bone resorption.
Cinacalcet	A type 2 calcimimetic agent, which increases the CaSR affinity for calcium, leading to PTH suppression and increased renal calcium excretion. It is used to increase the calcium sensitivity of mutated CaSR genes.

Abbreviation: CaSR, calcium-sensing receptor.

On arrival at the tertiary pediatric intensive care unit 17 hours after initial presentation, calcitonin 2 units/kg subcutaneous injection 6 hourly, and calcimetic, cinacalcet 2 mg/kg daily were commenced and frusemide increased to 6 hourly dosing. Low-calcium formula (Locasol) was introduced and IV fluids were continued with the total fluid intake equal to a double maintenance rate. Hemodialysis was considered but not required.

Calcium levels improved initially but rose whenever breastfeeds were introduced and total fluid volume reduced (Fig. 3). This was managed by increasing the cinacalcet to 2 mg/kg every 8 hours. The calcium level reduced; however, by day 14 of life had increased again to 4.2 mmol/L (16.83 mg/dL). At this point, IV fluids were increased to a double maintenance rate, whereas Locasol feeds were continued at 100 mL/kg/day. Frequency of frusemide and prednisone were additionally increased (Fig. 3). Because of the continued resistance to medical management, a surgical opinion was sought.

Total parathyroidectomy was performed on day 16 of life without complication.

**Figure 4.**
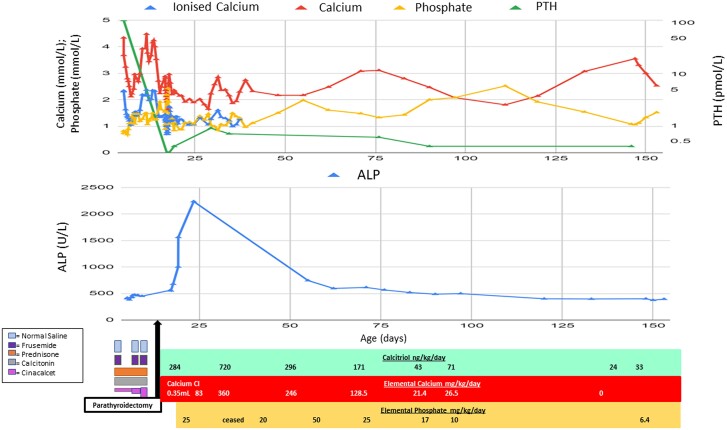
Blood test results and timeline of interventions including medications and respective doses.

It was anticipated that the baby would develop postoperative hypocalcemia, and hence a central line was inserted in the operating room and calcitriol 284 ng/kg/day commenced preemptively (Fig. 4). Despite this, there was an immediate drop in calcium (ionized calcium 1.44 mmol/L [5.77 mg/dL], serum calcium 2.36 mmol/L [16.83 mg/dL]), phosphate (0.9 mmol/L) (2.79 mg/dL), and PTH (4.8 pmol/L) (45 pg/mL). Approximately 10 hours following surgery, ionized calcium dropped to 0.70 mmol/L (2.81 mg/dL); therefore, IV calcium gluconate was given and an IV calcium infusion was continued for 3 days. Following this, oral calcium supplementation was introduced at 150 mg of elemental calcium twice daily. Breastfeeding was resumed, though because of difficulties establishing feeding, fatigue, and slow weight gain, nasogastric feeds were given until suck coordination improved. Postoperative calcium changes were asymptomatic.

On day 5 postoperatively, a progressively down-trending phosphate level was noted in addition to an increasing alkaline phosphatase, indicating increased bone turnover consistent with hungry bones syndrome (HBS). Other bone turnover markers were not measured; however, it was anticipated that they may have been elevated in the newborn period (4). Phosphate supplementation was commenced when the phosphate was 0.87 mmol/L (2.69 mg/dL) and alkaline phosphatase 1856 U/L (30.9 µkat/L) (120-550 U/L or 2-9.17 µkat/L). The HBS persisted for approximately 70 days following parathyroidectomy (Fig. 4). In this time, calcium and calcitriol were administered away from feeds to reduce the risk of inadequate absorption.

Given the neonate's lifelong need for calcium and calcitriol supplementation, concern was raised about the potential development of nephrocalcinosis (though urinary calcium excretion is low in this condition). Twenty days following surgery, urine calcium and urine calcium to creatinine ratio were undetectable (<0.3) and renal ultrasound showed no evidence of nephrocalcinosis.

On day 22 after surgery, electrolytes appeared stable and medication doses had remained unchanged. The neonate was transferred back to the peripheral hospital, and ongoing endocrinology advice was provided via telephone.

## Outcome and Follow-up

Macroscopically, all 4 parathyroid specimens were markedly increased in size for age (largest, 42 mg). The combined weight of 4 normal parathyroid glands for males aged 1 day to 3 months is 6.2 mg (5). Using frozen and paraffin sections, the glands were almost entirely parathyroid tissue on microscopy and distinguished from thymus by the lack of admixed adipose tissue with surrounding fibrous tissue. There were no atypical microscopic features within the parathyroid tissue.

PTH levels remain unmeasurable, in keeping with a successful complete parathyroidectomy. Calcium, calcitriol, and phosphate supplementation continued with phosphate being ceased 5 months after surgery.

The child is now 3 years old and has not required further hospitalization. He is thriving (height and weight tracking along 50th-75th percentile for age) and developing normally and continues to be monitored by his local pediatrician. He is monitored with monthly blood tests and has regular contact with his endocrinologist for titration of his calcium and calcitriol doses, which have reduced significantly from the immediate postoperative period (maximum calcitriol, 720 ng/kg/day 14 days postoperatively, down to most recently 28 ng/kg/day age 3 years).

## Discussion

NSHPT is a rare disorder consisting of hyperparathyroidism and marked hypercalcemia because of inactivation of the *CASR* gene. The condition can be fatal if not aggressively treated. Among reported cases, serum calcium and PTH levels vary widely and a more severe hypercalcemia is thought to result from homozygous variants (1-3, 6). A large case series of 57 cases (1) exploring genotype and phenotype reported only individuals with homozygous variants had calcium levels exceeding 4.5 mmol/L (18.04 mg/dL). However, counter to this expectation, our case was compound heterozygous for likely pathogenic variants in the *CASR* gene, with a serum calcium peaking at 5.4 mmol/L (21.64 mg/dL) and PTH levels higher than often found in infants with homozygous variants.

It is unclear from the existing literature if the underlying genetic cause of NSHPT can predict the response to medical management and necessity for surgical intervention, though there is some suggestion of correlation between responsiveness to bisphosphonates and calcimimetics and the individual variant involved (7, 8). Some compound heterozygous variants in the CaSR have been successfully managed with a type II calcimimetic drug (9), whereas others have required parathyroidectomy. Our case used all available medical options but hypercalcemia recurred when breast feeds were reintroduced, and parathyroidectomy was required.

Although surgery was immediately effective in reducing calcium and PTH levels, it precipitated HBS, a well-described phenomenon in patients who undergo parathyroidectomy, where total serum calcium concentration falls rapidly and hypocalcemia persists with associated hypophosphatemia (10). It is unclear, however, how frequently this occurs in patients with NSHPT after parathyroidectomy. In hyperparathyroidism, hypercalcemia occurs because of increased bone turnover, increased osteoclastic bone activity, and increased calcium reabsorption in the kidneys. Once the parathyroid gland is removed, the excessive osteoclastic activity is terminated and replaced by osteoblastic activity, which requires immediate bone calcium uptake for new bone formation, with resultant hypocalcemia (10). Serum phosphate levels decrease for the duration of the syndrome and alkaline phosphatase, which is involved in bone formation, may remain elevated for months (11), as was seen in our patient. Treatment of HBS requires aggressive supplementation of calcium, calcitriol, and phosphate, though phosphate replacement should be given cautiously as it may worsen hypocalcemia (10, 11). In the case of the infant described, his calcium requirements increased to 360 mg/kg/day and calcitriol 720 ng/kg/day, which, as a total daily dose, exceeds requirements of some adults with hypoparathyroidism.

Infants with NSHPT should have low urinary calcium excretion because of their underlying defect in the CaSR (1, 3), and thereby should not be at high risk for developing nephrocalcinosis during calcitriol and calcium replacement following parathyroidectomy (12). Hence, it may be possible to target a normal range serum calcium during replacement in those with NSHPT, rather than the lower end of the normal range in primary hypoparathyroidism (13).

In conclusion, this case highlights the complexities and challenges associated with managing patients with NSHPT including the initial clinical manifestations of hypercalcemia, difficulty predicting responsiveness to medical treatment, indications for surgical intervention, interpreting the significance of the particular genetic variant identified, and managing HBS with prolonged calcium, calcitriol, and phosphate supplementation.

## Learning Points

- Neonatal severe hyperparathyroidism (NSHPT) is a rarely occurring life-threatening disorder associated with variants in the calcium-sensing receptor (*CASR*) gene. It results in unopposed PTH activity that leads to skeletal demineralization, decreased renal calcium excretion, and persistent hypercalcemia.
- Clinically, neonates can present with a variety of signs.
- Treatment involves implementation of medical management and parathyroidectomy is indicated if medical management fails.
- The underlying genetic variant responsible for NSHPT does not necessarily predict severity of disease or likely responsiveness to treatment.

## Data Availability

Data sharing is not applicable to this article as no datasets were generated or analyzed during the current study.
